# Altitude Optimization and Task Allocation of UAV-Assisted MEC Communication System

**DOI:** 10.3390/s22208061

**Published:** 2022-10-21

**Authors:** Shuqi Huang, Jun Zhang, Yi Wu

**Affiliations:** 1 Fujian Provincial Engineering Technology Research Center of Photoelectric Sensing Application, Fujian Normal University, Fuzhou 350007, China; 2 Jiangsu Key Laboratory of Wireless Communications, Nanjing University of Posts and Telecommunications, Nanjing 210003, China

**Keywords:** UAV communication, mobile edge computing, delay, UAV altitude, task allocation

## Abstract

Unmanned aerial vehicles (UAVs) are widely used in wireless communication systems due to their flexible mobility and high maneuverability. The combination of UAVs and mobile edge computing (MEC) is regarded as a promising technology to provide high-quality computing services for latency-sensitive applications. In this paper, a novel UAV-assisted MEC uplink maritime communication system is proposed, where an MEC server is equipped on UAV to provide flexible assistance to maritime user. In particular, the task of user can be divided into two parts: one portion is offloaded to UAV and the remaining portion is offloaded to onshore base station for computing. We formulate an optimization problem to minimize the total system latency by designing the optimal flying altitude of UAV and the optimal task allocation ratio. We derive a semi closed-form expression of the optimal flying altitude of UAV and a closed-form expression of the optimal task allocation ratio. Simulation results demonstrate the precision of the theoretical analyses and show some interesting insights.

## 1. Introduction

With the rapid development of maritime activities such as marine tourism, oceanic mineral exploration, and maritime rescue, reliable and high-speed maritime wireless communication is receiving more and more attention [1]. In recent years, the use of intelligent applications has been rapidly popularized with the evolution of Internet of Things (IoT) [2,3,4]. The emergence of these latency-sensitive and compute-intensive applications, as well as the growth of maritime activities, has greatly improved the demand for maritime wireless communication. However, the satellite communication systems and high frequency/very high frequency (HF/VHF) communications used in traditional maritime communication networks have certain limitations [5,6]. Although satellite communications have a wide coverage area, they also have disadvantages such as high cost and large transmission delay. HF/VHF communication systems have lower costs, but they are not suitable for transmitting large amounts of data services due to insufficient channel bandwidth.

To tackle the above issues, mobile edge computing (MEC) can be an effective solution to provide high-quality computing services for compute-intensive maritime terminals. MEC places computing resources at the edge of the network, which can effectively reduce latency and significantly improve quality of service [7,8,9,10]. Many research works on MEC technology have received increasing attention. In [11], the task delay among users was minimized to optimize the tasks division ratios and offloading the transmit power. The authors in [12] proposed a MEC task offloading problem in ultra-dense networks, aiming to minimize latency while saving battery life of user devices. The offloading decision, user transmit power, and computation resource allocation were jointly optimized in [13] to minimize the weighted sum of energy consumption. In [14], the authors minimized the overall cost of energy, computation, and delay for all users by jointly optimizing the offloading decisions of all users’ tasks as well as the allocation of computation and communication resources. The authors in [15] minimized the sum of weighted task latency for all users by optimizing offloading decision, transmission power, and resource allocation. However, the above works in the field of MEC networks ignore a fact that the deployment of traditional MEC servers is fixed. In addition, due to the limited coverage of the ground MEC server, it is difficult for ground MEC to meet the computing requirement when the user devices are mobile.

In view of the above deficiencies, unmanned aerial vehicles (UAVs) can be regarded as a prospective solution. As a highly flexible aircraft, UAVs have been widely used in maritime wireless communication due to their advantages of high maneuverability, high scalability, and convenient deployment [16,17,18]. Hence, UAV has been proposed as an aerial computing platform to provide computing services for user devices, which is called UAV-assisted MEC. The use of UAV-assisted MEC can yield significant advantages over traditional ground MEC in terms of coverage, operational cost, and flexibility. Furthermore, line-of-sight (LoS) links from UAV to ground mobile users can also mitigate the impact of obstacles and shadows [19,20]. As a result, the combination of UAV and MEC can effectively improve the performance of maritime communication networks.

For UAV-assisted MEC system, the authors in [21] formulated a joint optimization problem to maximize the sum of the maximum delay among all users at each time slot. In [22], the authors minimized the weighted-sum energy consumption of UAV and users by jointly optimizing the UAV trajectory and computation resource allocation. In [23], the authors jointly optimized the time slot size, users scheduling, computation resource allocation, and UAVs’ trajectories to investigate a minimum time of task completion in a UAV-aided MEC system. However, the above research works adopt partial offloading approach to offload computational tasks to the UAV for computation. Since the computational capacity of user and UAV is limited, it is impractical to offload computational tasks to the UAV when the computational tasks are large. The work in [24] considered a UAV-enabled MEC system in which the UAV can act as an MEC server for computation or offload the tasks to base stations (BSs) for computation. It is worth noting that the LoS channel was adopted in [24], which ignored the effect of obstacle blockage in urban scenarios. Different from this paper, we use probabilistic channel that contain both LoS and non-LoS (NLoS) links, which makes our research more consistent with realistic scenarios.

The flying altitude of the UAV is an important issue, which is the focus of UAV-assisted wireless communication networks. However, the above existing works assume that the UAV flies at a fixed altitude without considering the optimization of the flying altitude. The work in [25] mainly focused on optimizing flying altitude and the number of UAVs to minimize a total power consumption. The altitude optimization problem of UAV based ad-hoc network was considered through minimizing the average network outage probability in [26]. For mixed radio frequency and underwater optical communication system, the authors determined an optimal altitude of the UAV by minimizing the system outage probability in [27]. However, most of the existing works in UAV-assisted MEC system have not investigated closed-form solution for the optimal flying altitude of the UAV. In addition, the above works have made significant contributions in the field of UAV-assisted MEC communication, but UAV-assisted MEC in maritime communication has not yet received wide attention.

In practice, UAV-assisted MEC maritime communication system investigated in our paper is meaningful. Firstly, the flexible mobility of UAV compensates the disadvantage of poor channel quality between ground MEC servers and users. Second, our research has certain potential to be applied in practical scenarios. For example, for maritime environments where network infrastructure is scarce and generated data are difficult to collect and monitor, using UAV as an MEC server can help maritime user process the generated compute-intensive data. Finally, we employ onshore BS and UAV to serve maritime user simultaneously, which can alleviate the computational burden of the network and further improve the computational performance of the system.

In this paper, we consider a maritime communication system in which a UAV equipped with an MEC server is used to provide computing services to maritime user. To better fit the reality, we adopt probabilistic channel between user and UAV, which is composed of both LoS and NLoS links. In addition to offloading computational tasks to UAV, the user can also offload to onshore BS with high computational capability for computation. The main contributions of this paper are summarised as follows.

- We propose a new UAV-assisted MEC framework in order to provide computing services for user with stringent latency requirement. In our proposed design, we formulate a task offloading problem for minimizing the total system delay to optimize the flying altitude of UAV and the task allocation ratio.
- To effectively solve the optimization problem, we derive a semi closed-form expression for the optimal flying altitude of the UAV and a closed-form expression for the optimal task allocation ratio, respectively.
- Simulation results demonstrate the accuracy of the analytical results and show that the optimal flying altitude depends on the horizontal distance between the UAV and the user. We also show the impacts of the transmit power, the bandwidth, and the CPU frequency on the optimal task allocation ratio.

The rest of this paper is organized as follows. We present the system model and the problem formulation for a UAV-assisted MEC uplink maritime communication system in Section 2. Then, we derive a semi closed-form expression of the optimal flying altitude of the UAV and a closed-form expression of the optimal task allocation ratio in Section 3. The simulation results are presented in Section 4 to confirm the accuracy of the theoretical results. Finally, we conclude the paper in Section 5.

## 2. System Model and Problem Statement

In this section, we present the system model and the formulation of the optimization problem. For ease of reference, the important notations in the system model are listed in Table 1.

### 2.1. System Model

**Figure 1:**
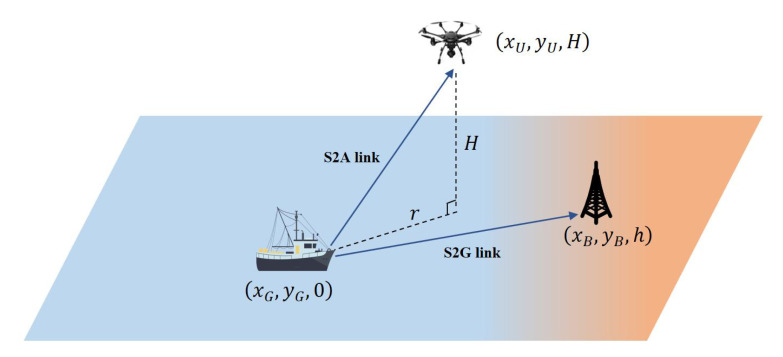
A UAV-assisted MEC uplink communication system.

As shown in Figure 1, we consider a UAV-assisted MEC uplink maritime communication system including a UAV, a maritime user, and an onshore BS, in which, they are all equipped with a single antenna. We use $\left(x_{U},y_{U},H\right)$, $\left(x_{G},y_{G},0\right)$, and $\left(x_{B},y_{B},h\right)$ to denote the three dimensional position of the UAV, the user, and the BS, respectively. We express the distance between the user and the projection of the UAV on the ground as $r=\sqrt{{\left(x_{U}-x_{G}\right)}^{2}+{\left(y_{U}-y_{G}\right)}^{2}}$. The user simultaneously offloads the tasks to the BS and the UAV for calculation based on frequency division multiple access (FDMA) scheme. The Sea-to-air (S2A) wireless communication link between user and UAV both contains LoS link and NLoS link [28]. The LoS probability of this link can be expressed as [29,30]

(1) $$p_{\mathrm{LoS}}=\frac{1}{1+a\exp[-b(\theta -a)]},$$

where *a* and *b* are constants determined by the environment, $\theta \;=\;\arctan(\frac{H}{r})$ denotes the elevation angle between UAV and the user. The probability of NLoS link between user and UAV is expressed as $p_{\mathrm{NLOS}}=1-p_{\mathrm{LoS}}$. The path loss of LoS link and NLoS link are as follows, respectively [31],

(2) $${\mathrm{PL}}_{\mathrm{LoS}}={\left(\frac{4\pi f_{c}d_{S2A}}{c}\right)}^{\varepsilon }\eta_{\mathrm{LoS}},$$

(3) $${\mathrm{PL}}_{\mathrm{NLoS}}={\left(\frac{4\pi f_{c}d_{S2A}}{c}\right)}^{\varepsilon }\eta_{\mathrm{NLoS}},$$

where *c* denotes the speed of light, $f_{c}$ denotes the carrier frequency, $d_{S2A}\;=\;\sqrt{H^{2}+r^{2}}$ is the distance between the UAV and the user, ε denotes the path loss exponent, $\eta_{\mathrm{LoS}}$ and $\eta_{\mathrm{NLoS}}$ denote the extra path loss of LoS link and NLoS link, respectively, and we set ε=2 [32,33]. The average path loss of S2A channel is expressed as follows

(4) $$\bar{\mathrm{PL}}=p_{\mathrm{LoS}}{\mathrm{PL}}_{\mathrm{LoS}}+p_{\mathrm{NLoS}}{\mathrm{PL}}_{\mathrm{NLoS}}.$$

The Sea-to-Ground (S2G) wireless communication link between user and BS is modeled as the Rayleigh fading channel [34], and the channel gain is expressed as

(5) $$h_{S2G}=\beta_{0}{\left(d_{S2G}\right)}^{-\varphi }\xi_{S2G},$$

where $\beta_{0}$ is the channel gain at the reference distance of 1 m, $d_{S2G}$ represents the distance between the user and the BS, φ denotes the path loss exponent, and we set φ=3.5, $\xi_{S2G}$ denotes the small-scale fading component conforming to the Rayleigh fading model.

The achievable rate of the uplink transmission task from the user to the UAV is given as

(6) $$R_{\mathrm{UAV}}=B_{\mathrm{UAV}}\log_{2}\left(1+\frac{PG}{\bar{\mathrm{PL}}N_{0}B_{\mathrm{UAV}}}\right),$$

where $B_{\mathrm{UAV}}$ represents the bandwidth allocated by the user to offload tasks to the UAV, *P* is the transmit power of the user, and $N_{0}$ is the spectral density of noise power. *G* denotes the gain of the directional antenna [35], it can be described as

(7) $$G=\left\{\begin{array}{c} \frac{G_{0}}{{\left(\frac{\omega }{2}\right)}^{2}},\;0\leq \phi \leq \frac{\omega }{2},\\ g\approx 0,\;otherwise \end{array}\right.$$

where ω denotes the beamwidth of UAV’s directional antenna, $G_{0}=30,000/2^{2}\;\times \;{\left(\pi /180\right)}^{2}\approx 2.2846$, ϕ denotes the sector angle, *g* is the channel gain beyond the beamwidth of the directional antenna. The achievable rate of the uplink transmission task from the user to the BS can be expressed as

(8) $$R_{\mathrm{BS}}=B_{\mathrm{BS}}\log_{2}\left(1+\frac{Ph_{S2G}}{N_{0}B_{\mathrm{BS}}}\right),$$

where $B_{\mathrm{BS}}$ denotes the bandwidth allocated by the user to offload the task to the BS.

Let *L* denotes the total computing tasks, the task allocation ratio is described as α∈[0,1]. The task allocation ratio from the user to the UAV and BS is α and 1−α, respectively. The total delay of the system is divided into two parts, one is the delay of transmission from the user offloading task to the UAV on the S2A link and the computation delay on the UAV, the other part is the transmission delay of the user offloading task to the BS on the S2G link and the computation delay on the BS.

The transmission delay of the user on S2A and S2G links can be, respectively, expressed as

(9) $$t_{S2A}=\frac{\alpha L}{R_{\mathrm{UAV}}},$$

(10) $$t_{S2G}=\frac{(1-\alpha )L}{R_{\mathrm{BS}}}.$$

The computation delay for the user processing tasks on the UAV and the BS can be, respectively, expressed as

(11) $$t_{\mathrm{UAV}}=\frac{\alpha LC}{f_{\mathrm{UAV}}},$$

(12) $$t_{\mathrm{BS}}=\frac{(1-\alpha )LC}{f_{\mathrm{BS}}},$$

where *C* represents the number of cycles required to compute one bit task, $f_{\mathrm{UAV}}$ is the CPU frequency allocated for computation on the UAV, and $f_{\mathrm{BS}}$ is the CPU frequency allocated for computation on the BS. The total system delay can be described as

(13) $$T=\max\left\{t_{S2A}+t_{\mathrm{UAV}},t_{S2G}+t_{\mathrm{BS}}\right\}.$$

### 2.2. Problem Statement

Based on the above discussion, we formulate a new optimization problem that minimizes the total delay of the system by optimizing the optimal flying altitude of the UAV and the optimal task allocation ratio. Specifically, the optimization problem can be expressed as follows

(14a) $$\begin{array}{cc} \; & \;\min_{\left\{H,\alpha \right\}}T \end{array}$$

(14b) $$\begin{array}{cc} \; & s.t.0\leq \alpha \leq 1, \end{array}$$

(14c) $$\begin{array}{cc} \; & \;H_{\min}\leq H\leq H_{\max}, \end{array}$$

where inequality (14b) represents the constraint of task allocation ratio, and inequality (14c) is the flying altitude constraint of the UAV.

## 3. Altitude of UAV and Task Allocation Optimization

In this section, the main results are presented to solve the problem of (14a). We derive a semi closed-form expression of the optimal altitude of the UAV and a closed-form expression of the optimal task allocation ratio, which are shown in the following results.

**Theorem** **1.** *According to optimization problem (14a), the optimal flying altitude of the UAV can be expressed as*

(15) $$H^{opt}=\frac{-At(H)}{2\eta_{\mathrm{NLoS}}{\left[1+at\left(H\right)\right]}^{2}+2\left(\eta_{\mathrm{LoS}}-\eta_{\mathrm{NLoS}}\right)\left[1+at\left(H\right)\right]},$$

*where*

(16) $$A=\frac{180}{\pi }\left(\eta_{\mathrm{LoS}}-\eta_{\mathrm{NLoS}}\right)abr,$$

(17) $$t\left(H\right)=\exp\left[-b\left(\frac{180}{\pi }\arctan\left(\frac{H}{r}\right)-a\right)\right],$$

*the optimal task allocation ratio of the UAV is given by*

(18) $$\alpha^{opt}=\frac{\frac{1}{R_{\mathrm{BS}}}+\frac{C}{f_{\mathrm{BS}}}}{\frac{1}{R_{\mathrm{UAV}}}+\frac{C}{f_{\mathrm{UAV}}}+\frac{1}{R_{\mathrm{BS}}}+\frac{C}{f_{\mathrm{BS}}}}.$$

**Proof of Theorem 1.**  Our aim is to minimize the total system delay, which depends on the maximum value of the transmission and computation delay of both S2A and S2G links, i.e., $\min\max\left\{t_{S2A}+t_{\mathrm{UAV}},t_{S2G}+t_{\mathrm{BS}}\right\}$. On the one hand, in the case of low flying altitude *H*, due to the stronger path loss of NLoS than LoS, the average path loss increases, which makes the transmission delay of the S2A link increase, resulting in an increase of the total system delay. On the other hand, when the flying altitude *H* is higher, the rate of user uplink transmission tasks to the UAV decreases, which makes the transmission delay of the S2A link increase, resulting in an increase of the total system delay. Therefore, there exists an optimal *H* that makes $t_{S2A}+t_{\mathrm{UAV}}=t_{S2G}+t_{\mathrm{BS}}$. We denote $t_{S2A}+t_{\mathrm{UAV}}$ as $T_{1}$, and denote $t_{S2G}+t_{\mathrm{BS}}$ as $T_{2}$. Substituting (10) and (12) into $T_{2}$, we obtain $T_{2}=\frac{(1-\alpha )L}{R_{\mathrm{BS}}}+\frac{(1-\alpha )LC}{f_{\mathrm{BS}}}$, which is independent of *H*. Substituting the rate of the user’s uplink transmission task to UAV in (6) into (9), the transmission delay of the S2A link can be obtained, and the expression of $T_{1}$ is expressed

(19) $$T_{1}=\frac{\alpha L}{B_{\mathrm{UAV}}\log_{2}\left(1+\frac{PG}{QN_{0}B_{\mathrm{UAV}}g\left(H\right)}\right)}+\frac{\alpha LC}{f_{\mathrm{UAV}}},$$

where

(20) $$Q={\left(\frac{4\pi f_{c}}{c}\right)}^{2},$$

(21) $$s\left(H\right)=H^{2}+r^{2},$$

(22) $$g\left(H\right)=s\left(H\right)\left[\eta_{\mathrm{NLoS}}+\frac{\eta_{\mathrm{LoS}}-\eta_{\mathrm{NLoS}}}{1+at(H)}\right].$$

In order to find an optimal altitude $H^{opt}$, then according to (19), which is equivalent to minimizing g(H), the first order derivative with respect to *H* is obtained as

(23) $$g^{\prime }\left(H\right)=2\eta_{\mathrm{NLoS}}H+\frac{2(\eta_{\mathrm{LoS}}-\eta_{\mathrm{NLoS}})H}{1+at(H)}+\frac{At(H)}{{\left[1+at\left(H\right)\right]}^{2}}.$$

Let $g^{\prime }\left(H\right)$ equals zero, and since 1+at(H)>0 holds, we have

(24) $$2\eta_{\mathrm{NLoS}}\;H{\left[1+at\left(H\right)\right]}^{2}\;+\;2\left(\eta_{\mathrm{LoS}}\;-\eta_{\mathrm{NLoS}}\right)\;H\left[1+at\left(H\right)\right]+\;At\left(H\right)\;=\;0.$$

The optimal flying altitude of the UAV in (15) can be obtained by arranging the above formula.

Substituting (9) and (11) into $T_{1}$, we get $T_{1}=\alpha \left(\frac{L}{R_{\mathrm{UAV}}}+\frac{LC}{f_{\mathrm{UAV}}}\right)$, and $T_{1}$ is monotonically increasing with respect to α, while $T_{2}=\left(1-\alpha \right)\left(\frac{L}{R_{\mathrm{BS}}}+\frac{LC}{f_{\mathrm{BS}}}\right)$ is monotonically decreasing with respect to α. Then according to (13), *T* has the minimum value when satisfying $T_{1}=T_{2}$, and $T_{1}=T_{2}$ can be expressed as

(25) $$\frac{\alpha L}{R_{\mathrm{UAV}}}+\frac{\alpha LC}{f_{\mathrm{UAV}}}=\frac{(1-\alpha )L}{R_{\mathrm{BS}}}+\frac{(1-\alpha )LC}{f_{\mathrm{BS}}}.$$

By arranging the above formula, we can get the optimal task allocation ratio in (18). □

From Theorem 1, we can see that the optimal flying altitude of the UAV depends on the distance between the UAV’s projection and the user. Therefore, UAV can adjust its flying altitude in time according to the change of the horizontal distance between itself and the user. We also find that the optimal task allocation ratio is proportional to the bandwidth allocated to the UAV by the user. Moreover, the transmit power of the user and the bandwidth allocated to the BS also affect the optimal task allocation ratio.

## 4. Numerical Results

In this section, numerical results are given to verify the accuracy of the theoretical results. We set the distance between the projection of the UAV on the ground and the user is r=50 m, environmental parameters are a=9.61, b=0.16 [36]. The channel gain at the reference distance of 1m is $\beta_{0}\;=\;-30$ dB, the distance between the user and the BS is $d_{S2G}=25$ m. The bandwidth allocated by the user to offload tasks to the UAV and the BS are $B_{\mathrm{UAV}}=B_{\mathrm{BS}}=1$ MHz, the beamwidth of UAV’s directional antenna is $\omega \;=\;100^{\circ }$. The CPU frequency allocated for computation on the UAV and the BS are $f_{\mathrm{UAV}}\;=\;1$ GHz, $f_{\mathrm{BS}}=10$ GHz. The other simulation parameters are shown in Table 2. In Table 1, $\eta_{\mathrm{LoS}}=1$ and $\eta_{\mathrm{NLoS}}=20$ are the parameters determined by the environment [29,36], and we choose the frequency band of 2.4 GHz as the carrier frequency due to the extensive use in UAV products and related work [37,38].

By iterating (15), the optimal flying altitude $H^{opt}$ can be obtained as 57 meters, and then substitute the iterative $H^{opt}$ into (18), the optimal task allocation ratio $\alpha^{opt}$ can be obtained as 0.62.

**Figure 2:**
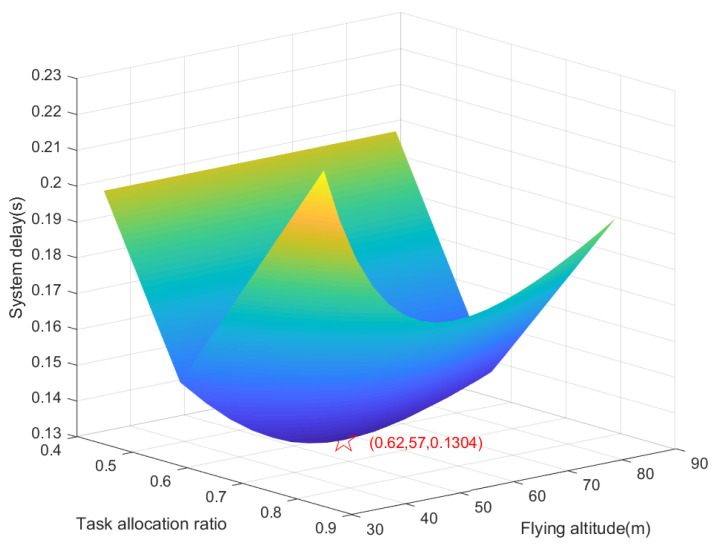
Total system delay versus task allocation ratio and flying altitude.

Figure 2 shows the total system delay versus the task allocation ratio and flying altitude of the UAV. We can see that the total system delay decreases and then increases as the task allocation ratio and flying altitude of the UAV increase, i.e., there exists an optimal flying altitude and optimal task allocation ratio that can minimize the total system delay, which is consistent with our analyses in Section 3.

**Figure 3:**
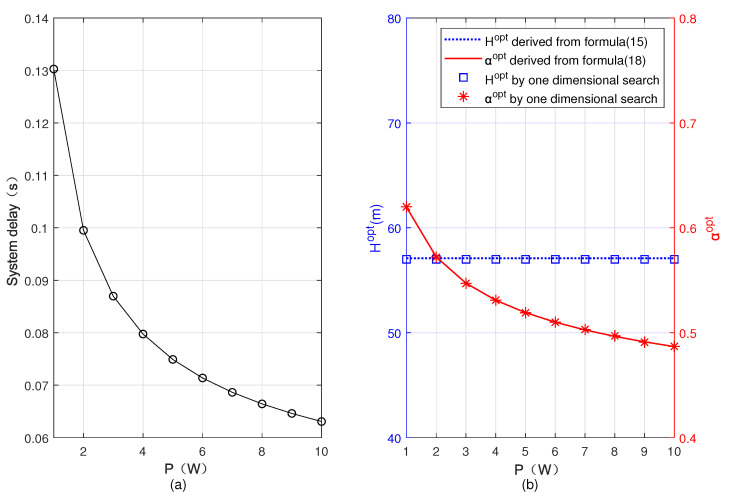
Minimum system delay, optimal flying altitude, and optimal task allocation ratio versus transmit power of the user. (**a**) Minimum system delay, (**b**) optimal flying altitude and optimal task allocation ratio.

Figure 3 shows the minimum system delay, $H^{opt}$, and $\alpha^{opt}$ versus *P*. It can be seen that as *P* increases, $\alpha^{opt}$ gradually decreases and the minimum total delay of the system also decreases. It is because the computing capability of UAV is weaker than that of BS, and when user’s transmit power increases, the user offloads more tasks to BS, and then the task allocation ratio of the user offloading tasks to UAV α decreases accordingly. We also see that with the increase of *P*, $H^{opt}$ remains unchanged. This is because the optimal flying altitude of the UAV is irrelevant to *P*. By comparing the results obtained based on (15) and (18) with the results obtained by one-dimensional search, we can observe that the two are basically identical.

**Figure 4:**
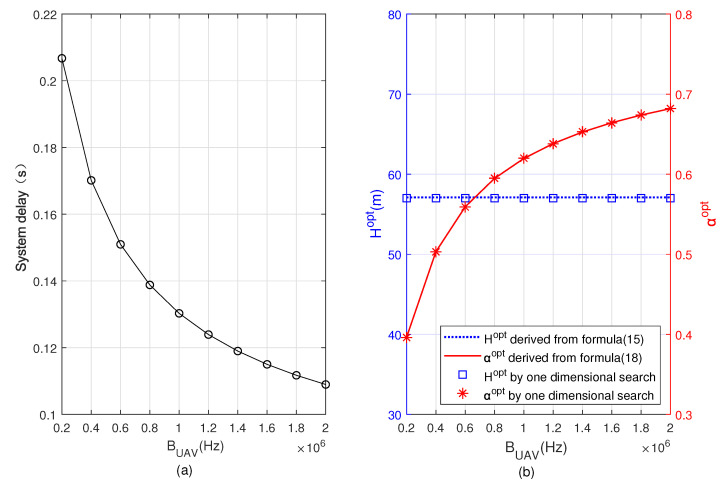
Minimum system delay, optimal flying altitude, and optimal task allocation ratio versus bandwidth allocated to UAV for task offloading. (**a**) Minimum system delay, (**b**) optimal flying altitude and optimal task allocation ratio.

**Figure 5:**
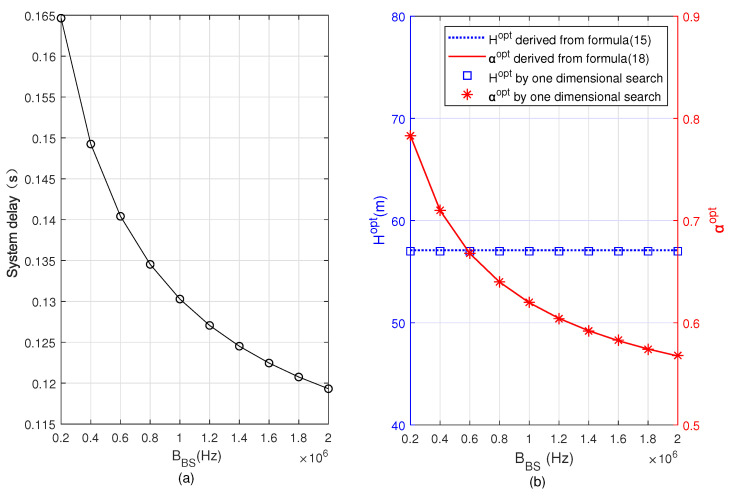
Minimum system delay, optimal flying altitude, and optimal task allocation ratio versus bandwidth allocated to BS for task offloading. (**a**) Minimum system delay, (**b**) optimal flying altitude and optimal task allocation ratio.

Figure 4 and Figure 5 depict the minimum system delay, $H^{opt}$, and $\alpha^{opt}$ versus $B_{\mathrm{UAV}}$ and $B_{\mathrm{BS}}$, respectively. Comparing Figure 4 and Figure 5, it can be observed that the minimum total delay of the system decreases with the increase of both $B_{\mathrm{UAV}}$ and $B_{\mathrm{BS}}$, $\alpha^{opt}$ increases in Figure 4 but decreases in Figure 5. It is because when $B_{\mathrm{UAV}}$ increases, the user offloads more computing tasks to the UAV, and the task allocation ratio of offloading to UAV α increases. However, when $B_{\mathrm{BS}}$ increases, the user offloads more computing tasks to the BS, and the task allocation ratio of offloading to BS 1−α increases, i.e., α decreases. In the two figures we can also see that $H^{opt}$ remains constant, since the optimal flight altitude is independent of $B_{\mathrm{UAV}}$ and $B_{\mathrm{BS}}$. The correctness of the analytical results can be verified by comparing the results based on Formulas (15) and (18) with the results obtained from the one-dimensional search.

**Figure 6:**
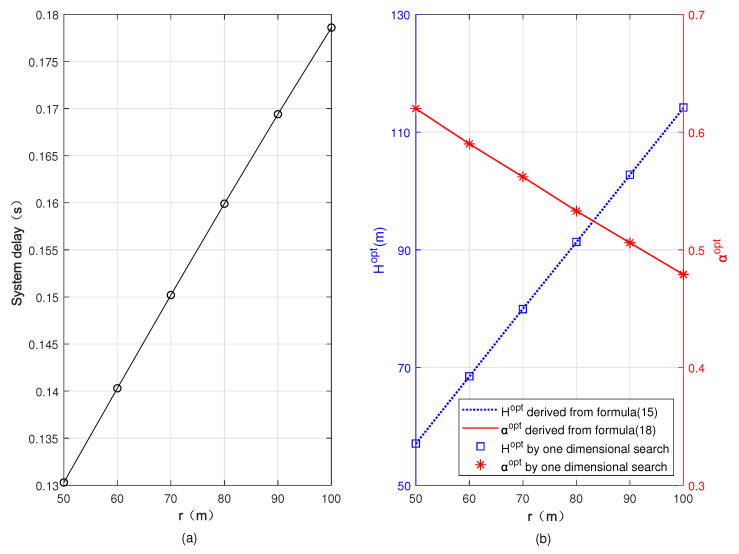
Minimum system delay, optimal flying altitude, and optimal task allocation ratio versus the distance between the UAV’s projection on the ground and the user. (**a**) Minimum system delay, (**b**) optimal flying altitude and optimal task allocation ratio.

**Figure 7:**
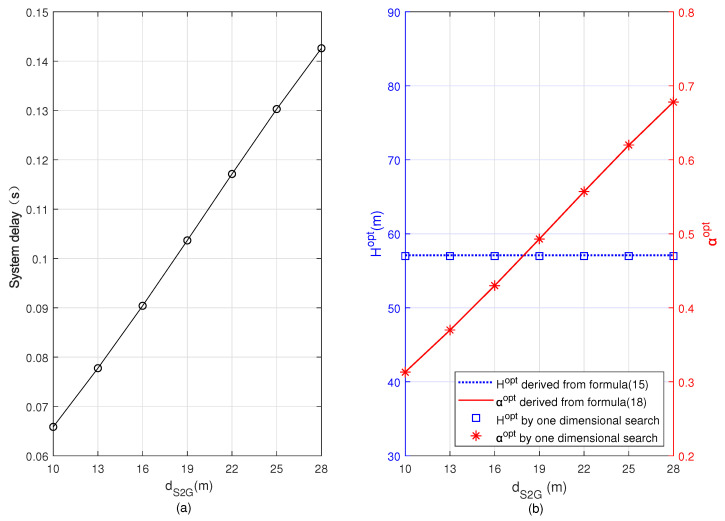
Minimum system delay, optimal flying altitude, and optimal task allocation ratio versus the distance between the user and the BS. (**a**) Minimum system delay, (**b**) optimal flying altitude and optimal task allocation ratio.

In Figure 6 and Figure 7, we plot the minimum system delay, $H^{opt}$, and $\alpha^{opt}$ versus *r* and $d_{S2G}$, respectively. From Figure 6, we can observe that with the increase of *r*, the minimum system delay and $H^{opt}$ increase, but $\alpha^{opt}$ decreases. The reason is that as *r* increases, the rate of the user’s uplink transmission to the UAV decreases, and the minimum total delay of the system increases accordingly. In order to minimize the total delay of the system, more computing tasks of the user are offloaded to the BS, so the task allocation ratio of offloading to the UAV α decreases. However, $\alpha^{opt}$ in Figure 7 increases with the increase of $d_{S2G}$. This is because the decrease of the transmission rate between the user and BS leads to an increase of the total system delay, and computing tasks will be more offloaded to the UAV in order to minimize the total system delay. We compare the results obtained based on Formulas (15) and (18) with the results obtained by one-dimensional search, and we can see that the results coincide basically, indicating the accuracy of our analyses in Section 3.

**Figure 8:**
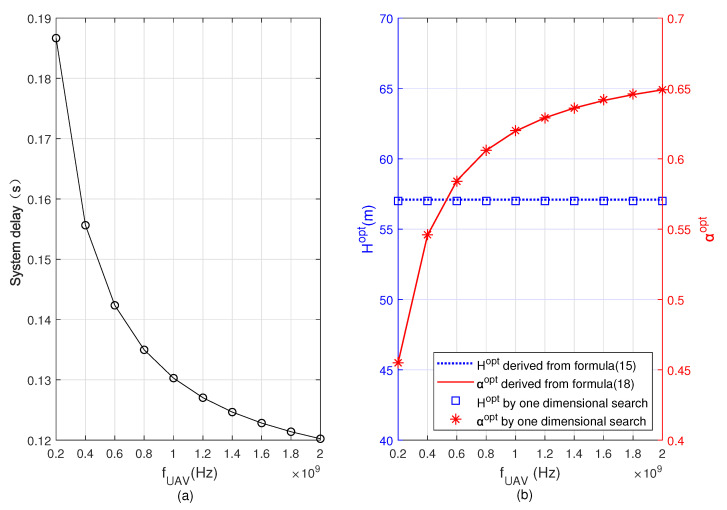
Minimum system delay, optimal flying altitude, and optimal task allocation ratio versus the CPU frequency allocated for computation on the UAV. (**a**) Minimum system delay, (**b**) optimal flying altitude and optimal task allocation ratio.

**Figure 9:**
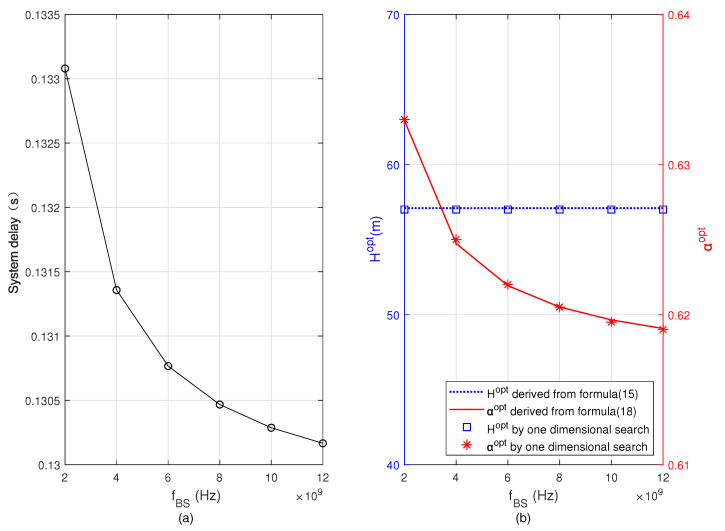
Minimum system delay, optimal flying altitude, and optimal task allocation ratio versus the CPU frequency allocated for computation on the BS. (**a**) Minimum system delay, (**b**) optimal flying altitude and optimal task allocation ratio.

Figure 8 and Figure 9 show the minimum system delay, $H^{opt}$, and $\alpha^{opt}$ versus $f_{\mathrm{UAV}}$ and $f_{\mathrm{BS}}$, respectively. By comparing Figure 8 and Figure 9, we see that when $f_{\mathrm{UAV}}$ and $f_{\mathrm{BS}}$ increase, respectively, the minimum total delay of the system decreases, $\alpha^{opt}$ gradually increases in Figure 8 but decreases in Figure 9. The reason is that when $f_{\mathrm{UAV}}$ increases, more computing tasks are offloaded to the UAV, then the task allocation ratio of task offloading to the UAV α increases. However, when $f_{\mathrm{BS}}$ increases, the user offloads more computing tasks to the BS, and the task allocation ratio of offloading to BS 1−α increases, i.e., α decreases. From the two figures, we also see that the optimal flying altitude $H^{opt}$ remains unchanged. This is because the optimal flying altitude of the UAV in Formula (15) is irrelevant to $f_{\mathrm{UAV}}$ and $f_{\mathrm{BS}}$. By comparing the results obtained based on Formulas (15) and (18) with the results obtained from the one-dimensional search, it can be seen that both are basically consistent.

## 5. Conclusions

In this paper, we investigated a UAV-assisted MEC uplink maritime communication system in which the UAV is employed as an MEC server to provide computing services to the user. In addition, the user can offload tasks to BS for computation. Our objective is to minimize the total delay of the system by optimizing the flying altitude of UAV and the task allocation ratio. The numerical results verified the effectiveness of the theoretical analyses and reveal that the optimal flying altitude of the UAV is determined by the horizontal distance between the UAV and the user. We also showed the relationship between the optimal task allocation ratio and system parameters such as the transmit power, the bandwidth, and the CPU frequency.

## Figures and Tables

**Table 1:** List of notations.

Symbol	Definition
*H*	Flying altitude of UAV
*T*	Total system delay
*c*	Speed of light
α	Task allocation ratio
*L*	Total computing tasks
*a*, *b*	Constants determined by the environment
θ	Elevation angle between UAV and the user
ω	Beamwidth of UAV’s directional antenna
*r*	Distance between the user and the projection of the UAV on the ground
ε, φ	Path loss exponent
$f_{c}$	Carrier frequency
$\eta_{\mathrm{LoS}}$, $\eta_{\mathrm{NLoS}}$	Extra path loss of LoS link and NLoS link
$p_{\mathrm{LoS}}$	LoS probability between the user and the UAV
$d_{S2A}$	Distance between the UAV and the user
$d_{S2G}$	Distance between the user and the BS
${\mathrm{PL}}_{\mathrm{LoS}}$, ${\mathrm{PL}}_{\mathrm{NLoS}}$	Path loss of LoS link and NLoS link
$\bar{\mathrm{PL}}$	Average path loss of S2A channel
$\beta_{0}$	Channel gain at the reference distance of 1m
$\xi_{S2G}$	Small-scale fading component
$h_{S2G}$	Channel gain of Rayleigh fading
*P*	Transmit power of the user
*G*	Gain of the directional antenna
$N_{0}$	Spectral density of noise power
$B_{\mathrm{UAV}}$, $B_{\mathrm{BS}}$	Bandwidth allocated by the user to offload tasks to the UAV and the BS
$R_{\mathrm{UAV}}$, $R_{\mathrm{BS}}$	Achievable offloading rate from the user to the UAV and the BS
$t_{S2A}$, $t_{S2G}$	Transmission delay of the user on S2A and S2G links
$f_{\mathrm{UAV}}$, $f_{\mathrm{BS}}$	CPU frequency allocated for computation on the UAV and the BS
$t_{\mathrm{UAV}}$, $t_{\mathrm{BS}}$	Computation delay for the user processing tasks on the UAV and the BS

**Table 2:** Simulation parameters.

Description	Value
LoS link extra path loss	$\eta_{\mathrm{LoS}}=1$
NLoS link extra path loss	$\eta_{\mathrm{NLoS}}=20$
Carrier frequency	$f_{c}=2.4$ GHz
Small-scale fading component	$\xi_{S2G}=0.7$
Noise power spectral density	$N_{0}=5\times 10^{-15}$ W/Hz
Total computing task	$L=5\times 10^{5}$ bit
Cycle for one bit	C=100 cycle/bit
Speed of light	$c=3.0\times 10^{8}$ m/s
Transmit power of the user	P=1 W

## Data Availability

Data available on request.
